# Intracardiac echocardiography for left ventricular diastolic function assessment during atrial fibrillation ablation

**DOI:** 10.1007/s10840-025-02110-y

**Published:** 2025-08-27

**Authors:** Jian Liang Tan, Julio Chirinos, Darshak Patel, Dinesh Jagasia, Matthew C. Hyman, Gustavo S. Guandalini, Saman Nazarian, David Lin, Gregory Supple, David S. Frankel, David J. Callans, Francis E Marchlinski, Jian-Fang Ren, Timothy M. Markman

**Affiliations:** 1 Section for Electrophysiology, Perelman School of Medicine, University of Pennsylvania, Philadelphia, PA USA; 2 Division of Cardiovascular Medicine, Perelman School of Medicine, University of Pennsylvania, Philadelphia, PA USA

**Keywords:** Atrial fibrillation, Diastolic function, Intracardiac echocardiography, Left atrial pressure, Transthoracic echocardiography, Outcome

## Abstract

**Background:**

Left ventricular (LV) diastolic dysfunction is associated with the development of atrial fibrillation (AF) and risk of recurrence after ablation. The use of an intracardiac echocardiography (ICE) for diastolic function assessment during ablation procedures has not been evaluated.

**Objectives:**

To evaluate the feasibility and utility of ICE obtained measures of LV diastolic function including peak tricuspid regurgitation velocity, trans-mitral flow velocity, mitral annular tissue Doppler velocities, and pulmonary vein flow velocities in patients undergoing AF ablation.

**Methods:**

We conducted a single-center, prospective evaluation of patients undergoing AF ablation between 2022 and 2024. During sinus rhythm, diastolic parameters were measured with the ICE catheter and direct left atrial pressure (LAP) was recorded prior to AF ablation. Elevated LAP was defined as ≥ 12 mmHg. ICE measured diastolic parameters were compared with those measured on transthoracic echocardiography (TTE).

**Results:**

A total of 152 patients (53% male, 69 ± 12 years old, mean CHA_2_DS_2_-VASc score of 3 ± 2) were analyzed, of which 80 had normal LAP (< 12 mmHg) by direct measurement. Several ICE parameters were found to be significantly associated with mean LAP, including greater peak tricuspid regurgitation velocity (*β* = 3.5; *p* = 0.005) and average E/e′ (*β* = 0.7; *p* < 0.001). In multivariable model, post-procedure intravenous diuretics were more commonly required in patients with abnormal diastolic function by ICE (mitral E/A OR = 8.1; average E/e′ OR = 24.2).

**Conclusions:**

ICE can be used to assess diastolic function with traditional parameters correlating with both TTE diastolic function and LAP. ICE measures of restrictive filling are associated with the need for post-procedural intravenous diuretics.

**Graphical Abstract:**

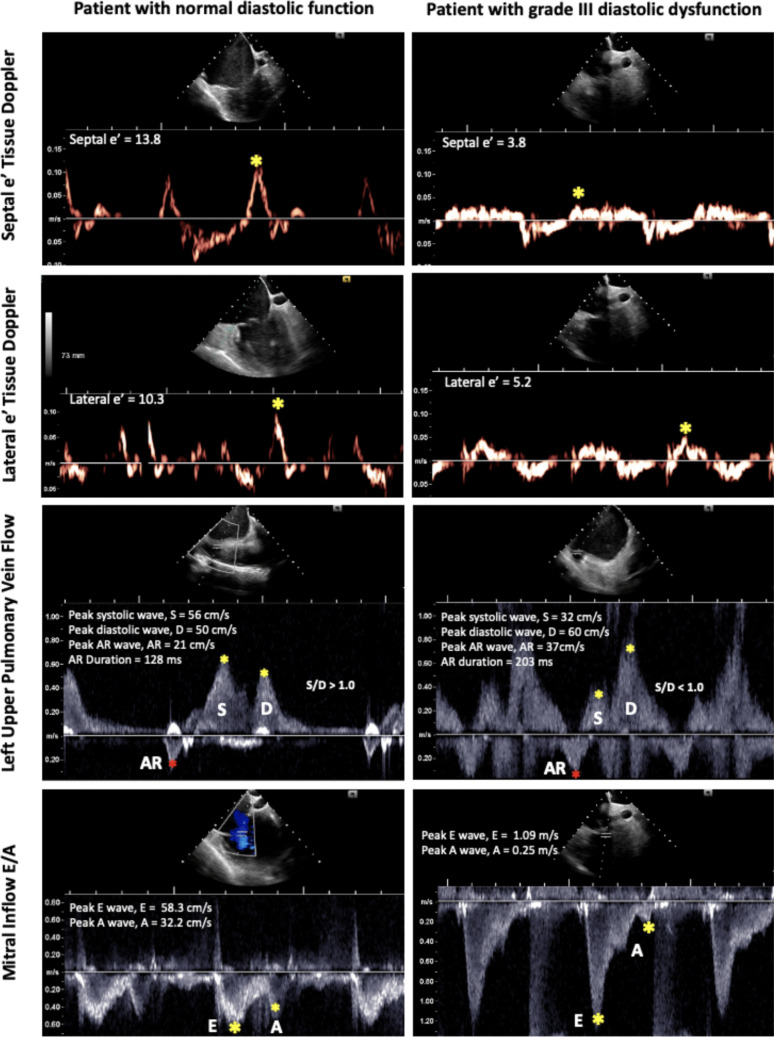

## Introduction

Diastolic function has a complex, bidirectional relationship with atrial fibrillation (AF). Abnormal diastolic function is associated with an increased risk for atrial fibrosis and AF. Conversely, the occurrence of AF can lead to further atrial fibrosis and diastolic dysfunction with elevation in left atrial pressure (LAP) [1, 2] and impaired atrial-ventricular coupling. Moreover, LAP may affect left atrial mechanical function through increased stretch and wall stress, resulting in progressive atrial remodeling in addition to symptoms of left sided volume and pressure overload [3–8]. Patients with both AF and diastolic dysfunction may be at higher risk of adverse cardiovascular events when compared to patients with either condition in isolation [9]. However, the assessment of diastolic function in AF remains challenging.

Transthoracic echocardiography (TTE) is commonly used to assess LV diastolic function and estimate LAP [10]. While all diastolic parameters are associated with left ventricular filling pressures, mitral annular E/A ratio and mitral annular velocity E/e′ ratio are particularly associated with restrictive ventricular filling, which can be present independent of acute, load-dependent filling pressures [11, 12]. Severe diastolic dysfunction may be a sign of restrictive cardiomyopathies, such as cardiac amyloidosis, which can initially present with atrial arrhythmias [8, 13]. Despite the value in assessment of LV diastolic function in patients with AF, TTE often fails to provide sufficient evaluation of diastolic function, either because of poor image quality, inadequate imaging, or the presence of AF at the time of the study, which limits the ability to interrogate relevant parameters.

Intracardiac echocardiography (ICE) has become a standard tool for AF ablation procedures, providing real-time information on cardiac anatomy to guide trans-septal access and catheter positioning during mapping and ablation and to monitor for acute procedural complications. Although it has become a standard of care for AF ablation, ICE has not been utilized to evaluate left atrial mechanical function and LV diastolic function in these patients. While LAP is less frequently directly measured in an era of wire-based left atrial access tools, estimation with ICE may help with volume management while additionally providing arrhythmic prognostication, or identification of co-existing restrictive pathology.

In this study, we aimed to (1) evaluate the feasibility of utilizing ICE to assess LV diastolic function parameters, (2) examine the relationship between ICE parameters of diastolic function and LAP and traditional TTE diastolic parameters among patients undergoing catheter ablation for AF, and (3) identify the association between ICE-derived diastolic parameters and the need for post-procedure intravenous diuretics.

## Methods

### Patient selection

A prospective single-center study was performed including consecutive patients who underwent catheter ablation for AF between 2022 and 2024 who had diastolic parameters assessed with ICE. Patients were excluded if sinus rhythm could not be maintained for ICE assessment prior to ablation. Diastolic parameters measured with the TTE study performed closest in time to the ablation procedure were also collected for comparison when patients had a TTE performed in sinus rhythm. All patients provided written informed consent for the ablation procedure, and the use of their data was approved by the Institutional Review Board of the University of Pennsylvania.

### Intracardiac echocardiography protocol

Following femoral venous access and prior to trans-septal access, an ICE catheter (SoundStar, 5–10 MHz, 64-element, Biosense Webster, Irvine, CA) was used in all cases to obtain the required echocardiographic measurements. For patients presenting for the procedure in AF, external cardioversion was performed to restore sinus rhythm prior to ICE measurements. All ICE measurements were made prior to performing any ablation. From the mid right atrium, the tip of the ICE catheter was positioned to view the tricuspid valve with maximal exposure of the color Doppler jet of tricuspid regurgitation. Peak tricuspid regurgitation velocity was measured using continuous wave Doppler. Clockwise rotation with the ICE catheter was then performed to identify the long axis view across the mitral valve with the cardiac apex in far-field at the distal aspect of the image. A relatively cranial position from the level of the right atrial/superior vena cava junction was often necessary with anteflexion to optimally align the angle of the image with the mitral inflow. Peak mitral E wave velocity and late A wave velocity were obtained from this view. Pulsed wave Doppler was used from this view, and a sample volume is placed between the mitral leaflet tips. Deceleration time was measured from the peak mitral E wave to when it reached baseline. From this view, a sample volume was placed over the basal septal and lateral mitral annulus for tissue Doppler recording (early diastolic velocities, e′) of the septal and lateral mitral annulus velocities. Further clockwise rotation of the ICE catheter was then performed to identify first the left and then the right pulmonary veins. Sample volume was placed approximately 1 mm into the ostium of the pulmonary veins for pulsed wave Doppler velocity measurements. LA diameter was measured in one view with the ICE transducer positioned in the RA from the fossa to the carina of the left pulmonary veins. All echocardiographic parameters were obtained to parallel recommendations from TTE and transesophageal echocardiography based on the American Society of Echocardiography (ASE) guidelines [10].

### Catheter ablation procedure

All patients underwent standard AF ablation under general anesthesia with direct arterial pressure and oxygen saturation monitoring. Decapolar catheters were placed in the coronary sinus and posterior right atrium. Immediately after the trans-septal puncture, the LAP was transduced directly via trans-septal sheath. Mean LAP was recorded in sinus rhythm. Elevated LAP was defined as mean LAP ≥ 12 mmHg. Ablation was then performed in all patients with radiofrequency energy using the 3.5-mm open-irrigated catheter (Thermocool SmartTouch SF, Biosense Webster). Ablation strategy utilized was at the discretion of the operator.

### LV diastolic function assessment

**Fig. 1:**
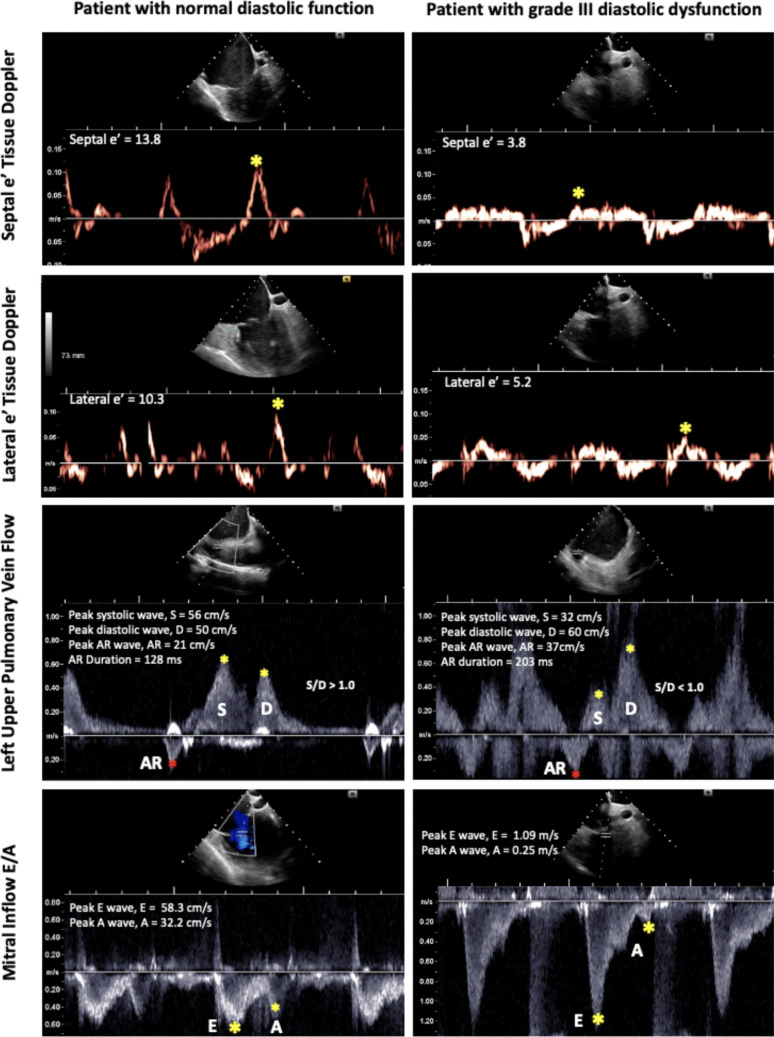
Intracardiac echocardiographic interrogation of left ventricular diastolic function in patients during AF ablation. A, peak mitral inflow velocity in late diastole; AR, atrial reversal; D, diastolic wave; e′, early diastolic mitral annular velocity using tissue Doppler imaging; E, peak mitral inflow velocity in early diastole; S, systolic wave

LV diastolic function was quantified in accordance with the ASE guidelines [11]. Measurements and diastolic function assessments were made off-line after the procedure by a cardiologist who was blinded to the left atrial pressure, TTE data, and need for post-procedure diuretics. As LA volume was not measured by the ICE catheter, the LA diameter, mitral E/A ratio, peak tricuspid regurgitation velocity, and average E/e′ were used to determine the LV diastolic function as discussed below. In brief, patients with normal estimated LAP based on established 2016 guidelines from the ASE and the European Association of Cardiovascular Imaging [10], mitral E/A ratio ≥ 0.8, average E/e′ < 10, and peak tricuspid regurgitation velocity < 2.8 m/s were categorized as normal LV diastolic function. Patients with grade I, II, or III LV diastolic function were categorized based on their respective ICE diastolic parameters and estimated LAP as shown in Table 1 and Fig. 1.

**Table 1:** Left ventricular diastolic function according to left atrial pressure and intracardiac echocardiogram parameters [11]

	**Normal**	**Grade I diastolic dysfunction**	**Grade II diastolic dysfunction**	**Grade III diastolic dysfunction**
Mitral E/A	≥ 0.8	< 0.8	≥ 0.8 to < 2.0	≥ 2.0
Average E/e′	< 10	< 10	10–14	> 14
Peak tricuspid regurgitation velocity (m/s)	< 2.8	< 2.8	≥ 2.8	≥ 2.8
Estimated left atrial pressure	Normal	Low or normal	Elevated	Elevated

### Statistical analysis

Categorical variables are expressed as counts with percentages and compared using the Pearson *χ*^2^ test or ANOVA. Continuous variables are expressed as mean ± standard deviation (SD) or median and interquartile range (IQR) and compared using *t*-tests. Non-parametric statistical hypothesis tests were used when data was not normally distributed. Correlation of the ICE diastolic parameters with mean LAP and TTE diastolic parameters was performed using linear regression analysis. Variables were screened for inclusion in a multivariable model based on univariate association (*p* < 0.10). Subsequently, variables meeting predefined criteria for entry (*p* < 0.05) were included in the final multivariable model, which was assessed for goodness-of-fit using the *R*^2^ value. Correlation of clinical factors and ICE diastolic parameters with postoperative intravenous diuretics was performed using logistic regression analysis. Variables were screened for inclusion in a multivariable model based on univariate association (*p* < 0.10). Subsequently, variables meeting predefined criteria for entry (*p* < 0.05) were included in the final multivariable model, which was assessed for strength of the model using McFadden’s *R*^2^ value with values ≥ 0.4 suggestive of a strong model. *p*-values < 0.05 were considered statistically significant. Statistical analyses were performed using Stata 12.4 (StataCorrp, College Station, TX, USA).

## Results

### Patient population

A total of 152 patients (53% male, 69 ± 12 years old, mean CHA_2_DS_2_-VASc score of 3 ± 2) met enrollment criteria. Baseline demographic and patient characteristics between patients with normal and elevated LAP by direct left atrial measure are summarized in Table 2. Of the 152 patients, 62 (41%) had a clinical diagnosis of heart failure, 99 (65%) had hypertension, 26 (17%) had diabetes mellitus, and 81 (53%) of patients had persistent AF. Seventy-one (47%) patients were in AF at the time of their ablation procedure, requiring cardioversion prior to ICE diastolic measurements. Patients with higher body mass index, hypertension, diabetes mellitus, and heart failure were more likely to have elevated LAP. Patients with persistent AF and those with baseline AF at the time of the ablation procedure were also more likely to have elevated LAP.

**Table 2:** Baseline clinical characteristics of patients. Continuous variables are reported as mean ± standard deviation, and categorical variables are reported as absolute count and percentage. *p*-values < 0.05 are noted in bold font

	All patients (*n* = 152)	Mean left atrial pressure < 12 mmHg (*n* = 80)	Mean left atrial pressure ≥ 12 mmHg (*n* = 72)	*p*-value
Age, years	68.9 ± 11.8	69.3 ± 9.7	67.9 ± 9.7	0.4
Male	81 (53%)	44 (55%)	37 (51%)	0.6
Body mass index, kg/m^2^	28.9 ± 6.3	27.7 ± 7.1	30.3 ± 6.7	**0.02**
CHA_2_DS_2_-VASC score	3.1 ± 1.3	2.9 ± 1.3	3.4 ± 1.7	0.05
Heart failure	62 (41%)	20 (25%)	42 (58%)	** < 0.001**
Coronary artery disease	30 (20%)	16 (20%)	14 (19%)	0.9
Prior stroke or transient ischemic attack	11 (7%)	5 (6%)	6 (8%)	0.7
Hypertension	99 (65%)	47 (59%)	52 (72%)	0.08
Diabetes mellitus	26 (17%)	7 (9%)	19 (26%)	**0.004**
Chronic kidney disease ≥ stage III	20 (13%)	9 (11%)	11 (15%)	0.6
Creatinine, mg/dL	1.3 ± 1.7	1.0 ± 0.9	1.3 ± 2.1	0.3
Persistent atrial fibrillation	81 (53%)	25 (31%)	56 (78%)	** < 0.001**
Baseline rhythm atrial fibrillation at time of ablation	71 (47%)	10 (13%)	61 (85%)	** < 0.001**
Failed antiarrhythmic drug	99 (65%)	44 (55%)	55 (76%)	**0.006**
Left ventricular ejection fraction, %	52.8 ± 12.1	55.1 ± 10.2	51.4 ± 14.8	0.08
Left atrial pressure, mmHg	11.7 ± 5.0	7.5 ± 2.2	16.0 ± 3.4	** < 0.001**

Diastolic parameters were able to be obtained in all patients and total time to collect all relevant ICE images was < 10 min in all patients. One patient developed pericarditis with a trivial post-ablation pericardial effusion that was treated conservatively. There were no other complications associated with the procedures.

### Correlation between intracardiac echocardiographic parameters and mean left atrial pressure

**Fig. 2:**
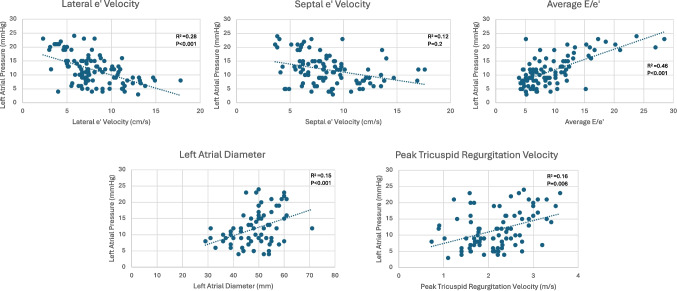
Linear regression of intracardiac echocardiographic parameters with directly measured mean left atrial pressure

Several ICE parameters were found to be significantly associated with mean LAP, including greater peak tricuspid regurgitation velocity (*β* = 3.5; *p* = 0.005), average E/e’ (β = 0.7; p < 0.001), and left atrial diameter (β = 0.2; p < 0.001). Additionally, multiple ICE parameters were found to be significantly different between patients with normal vs elevated mean LAP. The latter subgroup exhibited greater peak tricuspid regurgitation velocity (2.7 ± 0.5 vs 1.9 ± 1.0 m/s, p < 0.001), average E/e’ (13.1 ± 15.9 vs 6.7 ± 2.1, p < 0.001), and left atrial diameter (51.0 ± 7.8 vs 45.1 ± 8.6 mm, p < 0.001). Pulmonary venous S/D ratio and atrial reversal duration were also associated with LAP (Table 3). Univariable linear regression analyses demonstrated lateral mitral annular e’ velocity, average E/e', LA diameter, tricuspid regurgitation velocity, and E/A obtained using ICE catheter were independently associated with mean LAP (Fig. 2). All patients with elevated left atrial pressure had evidence of diastolic dysfunction detected by ICE (Table 3).

**Table 3:** Intracardiac echocardiographic and procedural characteristics. Continuous variables are reported as mean ± standard deviation and categorical variables are reported as absolute count and percentage. *p*-values < 0.05 are noted in bold font

	All patients (*n* = 152)	Mean left atrial pressure < 12 mmHg (*n* = 80)	Mean left atrial pressure ≥ 12 mmHg (*n* = 72)	*p*-value
**Diastolic parameters**				
Peak tricuspid regurgitation velocity, m/s	2.2 ± 0.9	1.9 ± 1.0	2.7 ± 0.5	**0.001**
Mitral Doppler E/A ratio				** < 0.001**
≤ 0.8	16 (11%)	16 (20%)	0	
> 0.8 to < 2.0	117 (77%)	61 (78%)	56 (77%)	
≥ 2.0	19 (13%)	3 (4%)	16 (22%)	
Average E/e**′**	9.6 ± 4.9	6.7 ± 2.1	13.1 ± 15.9	**0.002**
Left atrial diameter, mm	48.5 ± 8.1	45.1 ± 8.6	51.0 ± 7.8	** < 0.001**
Left superior pulmonary vein S/D ratio	1.1 ± 0.7	1.5 ± 0.6	0.9 ± 0.5	** < 0.001**
Left superior pulmonary vein atrial reversal duration, ms	106 ± 32	97 ± 24	118 ± 41	** < 0.001**
Left inferior pulmonary vein S/D ratio	1.1 ± 0.6	1.3 ± 0.5	0.9 ± 0.7	** < 0.001**
Left inferior pulmonary vein atrial reversal duration, ms	101 ± 35	100 ± 35	103 ± 36	0.6
Right superior pulmonary vein S/D ratio	1.1 ± 0.6	1.3 ± 0.5	0.8 ± 0.5	** < 0.001**
Right superior pulmonary vein atrial reversal duration, ms	105 ± 28	104 ± 22	109 ± 35	0.3
Right inferior pulmonary vein S/D ratio	1.2 ± 0.8	1.3 ± 0.6	1.1 ± 0.9	0.1
Right inferior pulmonary vein atrial reversal duration, ms	100 ± 28	96 ± 29	108 ± 28	**0.01**
**Diastolic function grade based on intracardiac echocardiography parameters**				** < 0.001**
Normal function	20 (13%)	20 (25%)	0	
Grade I dysfunction	47 (32%)	44 (55%)	3 (4%)	
Grade II dysfunction	64 (42%)	16 (20%)	48 (67%)	
Grade III dysfunction	21 (13%)	0	21 (29%)

In multivariable linear regression, lateral e′ velocity (*β* = −0.3, *p* = 0.008), average E/e*′* (*β* = 0.5, *p* < 0.001), and peak tricuspid regurgitation velocity (*β* = 1.9, *p* = 0.002) were associated with directly measured left atrial pressure (*R*^2^ = 0.63).

### Correlation between intracardiac echocardiographic parameters and transthoracic echocardiographic parameters

**Fig. 3:**
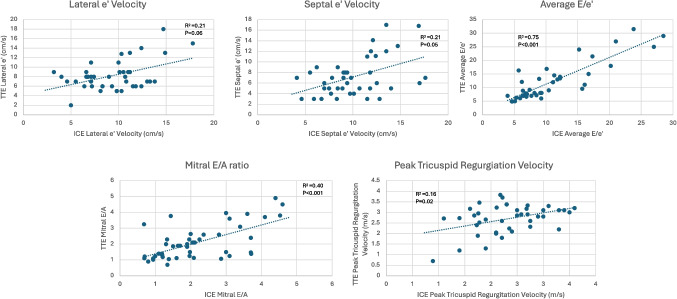
Linear regression of intracardiac echocardiographic parameters with transthoracic echocardiographic parameters. Abbreviations: ICE = intracardiac echocardiography; TTE = transthoracic echocardiography

By univariable linear regression, average E/e**′**, mitral E/A ratio, and peak tricuspid regurgitation velocity obtained utilizing intraprocedural ICE were significantly associated with the same parameters obtained on TTE (Fig. 3).

### Association between intracardiac echocardiographic parameters and post procedure intravenous diuretic requirements

Prior to discharge, 28 patients (18%) of patients required intravenous diuretics to manage volume overload (Table 4). Two additional patients, who were discharged without receiving intravenous diuretics, presented to the emergency room after discharge with volume overload requiring diuresis. Among these 30 patients, all had abnormal diastolic function while only 10 (33%) patients had elevated LA pressure at the time of direct measurement. Patients who required intravenous diuretics were more likely to have evidence on ICE of abnormal mitral E/A ratio ≥ 0.8 (100 vs 87%, *p* = 0.04), average E/e′ ≥ 10 (100 vs 14%, *p *< 0.001), peak tricuspid regurgitation velocity (40 vs 22%, *p* = 0.04), and elevated direct measurement of LA pressure ≥ 12 mmHg (33 vs 16%, *p* = 0.04).

**Table 4:** Clinical characteristics compared between patients who required and did not require post-procedural diuretics. Continuous variables are reported as mean ± standard deviation and categorical variables are reported as absolute count and percentage. *p*-values < 0.05 are noted in bold font

	All patients (*n* = 152)	Post-procedural diuretics required (*n *= 30)	No post-procedural diuretics (*n *= 122)	*p*-value
Age, years	68.9 ± 11.8	69.6 ± 12.1	68.7 ± 11.7	0.7
Male	81 (53%)	14 (47%)	67 (55%)	0.3
Body mass index, kg/m^2^	28.9 ± 6.3	29.3 ± 6.2	28.8 ± 6.4	0.4
CHA_2_DS_2_-VASC score	3.1 ± 1.3	3.4 ± 1.4	3.0 ± 1.2	0.19
Heart failure	62 (41%)	17 (57%)	45 (37%)	**0.01**
Coronary artery disease	30 (20%)	8 (27%)	22 (18%)	0.6
Prior stroke or transient ischemic attack	11 (7%)	3 (10%)	8 (7%)	0.2
Hypertension	99 (65%)	22 (73%)	77 (63%)	0.2
Diabetes mellitus	26 (17%)	7 (23%)	19 (16%)	0.2
Chronic kidney disease ≥ stage III	20 (13%)	6 (20%)	14 (11%)	0.08
Creatinine, mg/dL	1.3 ± 1.7	1.7 ± 2.2	1.2 ± 1.6	0.06
Persistent atrial fibrillation	81 (53%)	19 (63%)	62 (51%)	0.1
Baseline rhythm atrial fibrillation at time of ablation	71 (47%)	15 (50%)	56 (46%)	0.6
Failed antiarrhythmic drug	99 (65%)	20 (67%)	79 (65%)	0.3
Left ventricular ejection fraction, %	52.8 ± 12.1	47.4 ± 13.2	54.2 ± 11.4	**0.03**
Left atrial pressure, mmHg	11.7 ± 5.0	12.1 ± 5.2	11.6 ± 5.0	0.2
Duration of ablation procedure (minutes)	117 ± 32	125 ± 36	115 ± 31	0.09
Radiofrequency ablation time (minutes)	47 ± 15	50 ± 17	46 ± 14	0.1

On multivariable logistic regression, history of heart failure (OR = 2.3, *p* < 0.001), abnormal mitral E/A ratio (OR = 8.1, *p *< 0.001), and average E/e′ (OR = 24.2, *p *< 0.001) were significantly associated with the need for postoperative intravenous diuretics (McFaden’s *R*^2^ = 0.4).

## Discussion

Although ICE has several real-time clinical applications during AF ablation procedures that have made its use routine, this application has not been reported [14]. Given the ability to perform diastolic assessments with standard equipment utilized for ICE studies and the overlap between standard clinical views and those needed for AF ablation procedural guidance, real-time assessment of diastolic parameters may be easily obtained during routine AF ablations.

In this initial experience, the use of ICE for LV diastolic measurements during AF ablation was feasible and safe with the ability to accurately determine diastolic parameters in real time. No adverse events were related to the use of ICE catheter during AF ablation procedures, as was anticipated given overlap with standard ICE catheter positioning. It is similarly unsurprising that this assessment did not add significant time to the procedure with the entire assessment being completed in < 10 min for all patients.

Our data demonstrates ICE-derived parameters of diastolic function correlate well with both simultaneous direct measurements of left atrial pressure and traditional TTE diastolic parameters. Furthermore, they suggest that these parameters are highly correlated with post-procedural volume overload and the need for intravenous diuretics.

Given the value of understanding diastolic parameters, routine diastolic assessment is now performed using TTE when relevant parameters can be determined [4, 10, 15–17]. Unfortunately, determination of diastolic function can often be challenging or impossible especially in patients with persistent AF who may be in AF at the time of the study or in patients with body habitus that limit the quality of images. Although TTE is the standard tool for this assessment, the quality of the data obtained using ICE can be evaluated both by comparison to TTE and by correlation to direct LAP. Here, we found that several ICE parameters (lateral and septal e′, average E/e′, E/A, and LA diameter) correlated significantly with directly measured LAP. Additionally, ICE parameters and traditional parameters obtained using TTE (lateral E/e′, septal E/e′, average E/e′, and E/A) correlated well in LV diastolic assessment.

Previous studies have shown the importance of recognizing patients with elevated LAP and abnormal LV diastolic function in patients with AF [7, 18–21]. The ability to evaluate these parameters using ICE at the time of catheter ablation procedures has several potential implications including acute guidance of volume management at baseline or with serial measurements as LAP is less frequently measured in an era of wire-based left atrial access tools, arrhythmic prognostication guiding ablation or antiarrhythmic medication strategy, or identification of co-existing diastolic pathology including restrictive processes such as cardiac amyloidosis.

Given increased attention to the goal of same day discharge following AF ablation, it is increasingly valuable to identify patients who may require intravenous diuretics as volume overload may be a limiting factor in the ability to achieve this goal safely and effectively [22–25]. In our series, only three clinical or ICE-derived parameters were significantly associated with the need for intravenous diuretics in multivariable modeling. While clinical history of heart failure was associated, it had a modest odds ratio compared to ICE parameters of restrictive filling (mitral E/A and average E/e′). Similarly, measures of left ventricular systolic function and elevated filling pressures including direct pressure measurements were not significantly associated with intravenous diuretics in the multivariable model. This may be partially due to the more obvious need for cautious volume management in these patients, as opposed to a population of patients with normal ejection fraction and without elevated filling pressures at baseline, who may be particularly vulnerable to volume overload as a result of the fluid given during ablation. It is therefore patients with restrictive ventricular filling identified on ICE without other obvious risk factors who may benefit most significant from identifying a clear risk factor for acute procedural volume overload.

Prior data indicate that abnormal LV filling pressure may predict poor clinical outcomes in patients undergoing AF ablation [7, 19–21]. Patients with LV dysfunction pre-AF ablation may have increased risk for worsening heart failure symptoms, heart failure hospitalization, and AF recurrence post-AF ablation [18–21]. For these reasons, future studies should evaluate the association between ICE-derived diastolic parameters and arrhythmia recurrence as well as heart failure outcomes after AF ablation and whether ICE parameters obtained during the ablation procedure can predict these clinical outcomes independent of mean LAP at the time of ablation.

### Limitations

This study includes the typical limitations of a single-center prospective cohort study with relatively short-term analysis of clinical outcomes. Longer-term follow-up and larger sample size are warranted to verify the results in our study. Volume and pressure loads were not identical at the time of ICE and TTE measurements and may be impacted by the use of general anesthesia and mechanical ventilation, meaning that direct comparison of the ICE parameters with the TTE parameters might be limited. Intraprocedural diastolic function may be impacted by several factors including the effects of anesthesia, positive pressure ventilation, and fluid shifts, as well as changes in autonomic time and heart rate.

The technical limitations associated with the interrogation of mitral inflow and annular velocities with ICE should also be considered. In particular, proper alignment of the interrogation line with the mitral inflow and/or direction of annular motion is challenging with ICE. This may be particularly important for the longitudinal motion of the lateral annulus, which does not coincide with the interrogation line arising from the right atrial probe (Fig. 1), which likely leads to the underestimation of tissue velocities as we may see here given the lower lateral compared to septal velocities, which is less frequently seen with TTE. Future studies should assess strategies to overcome these limitations, including theory-based angle correction strategies and 2D-imaging-based automated tracking of base to apex mitral annular motion.

## Conclusions

Our study demonstrates the feasibility of ICE to assess left ventricular diastolic function and suggests a potential role in assessment of LV diastolic function, especially in patients with persistent AF during AF ablation. ICE-derived measures of restrictive filling, namely mitral E/A and average E/e′, were significantly associated with the need for post-procedural intravenous diuretics. Additional evaluation is necessary to determine the optimal criteria for diastolic dysfunction using ICE, to overcome some technical limitations of the technique, and to evaluate the association between intraprocedural diastolic parameters, arrhythmia recurrence, and heart failure outcomes.

## Abbreviations

AF: Atrial fibrillation
ASE: American Society of Echocardiography
ICE: Intracardiac echocardiography
LA: Left atrium
LAP: Left atrial pressure
LV: Left ventricular
LVEF: Left ventricular ejection fraction
PVI: Pulmonary vein isolation
TTE: Transthoracic echocardiography
